# A Deep Learning–Based Framework for Supporting Clinical Diagnosis of Glioblastoma Subtypes

**DOI:** 10.3389/fgene.2022.855420

**Published:** 2022-03-28

**Authors:** Sana Munquad, Tapas Si, Saurav Mallik, Asim Bikas Das, Zhongming Zhao

**Affiliations:** ^1^ Department of Biotechnology, National Institute of Technology Warangal, Warangal, India; ^2^ Department of Computer Science and Engineering, Bankura Unnayani Institute of Engineering, Bankura, India; ^3^ Center for Precision Health, School of Biomedical Informatics, The University of Texas Health Science Center at Houston, Houston, TX, United States; ^4^ Human Genetics Center, School of Public Health, The University of Texas Health Science Center at Houston, Houston, TX, United States; ^5^ Department of Pathology and Laboratory Medicine, McGovern Medical School, The University of Texas Health Science Center at Houston, Houston, TX, United States

**Keywords:** deep learning, glioblastoma multiforme, biomarkers, co-expression gene module, machine learning

## Abstract

Understanding molecular features that facilitate aggressive phenotypes in glioblastoma multiforme (GBM) remains a major clinical challenge. Accurate diagnosis of GBM subtypes, namely classical, proneural, and mesenchymal, and identification of specific molecular features are crucial for clinicians for systematic treatment. We develop a biologically interpretable and highly efficient deep learning framework based on a convolutional neural network for subtype identification. The classifiers were generated from high-throughput data of different molecular levels, i.e., transcriptome and methylome. Furthermore, an integrated subsystem of transcriptome and methylome data was also used to build the biologically relevant model. Our results show that deep learning model outperforms the traditional machine learning algorithms. Furthermore, to evaluate the biological and clinical applicability of the classification, we performed weighted gene correlation network analysis, gene set enrichment, and survival analysis of the feature genes. We identified the genotype–phenotype relationship of GBM subtypes and the subtype-specific predictive biomarkers for potential diagnosis and treatment.

## Introduction

Glioblastoma multiforme (GBM), which is the grade IV of glioma as defined by the World Health Organization (WHO), is a highly invasive and devastating primary form of brain cancer. The complexity and molecular heterogeneity of GBM pose the challenge for accurate diagnosis and therapy (Verhaak et al., 2010; Zhang et al., 2020). The prognosis for patients with GBM is poor, and median survival is 12 months (Witthayanuwat et al., 2018). Because of enormous molecular heterogeneity and difficulty in early diagnosis, the molecular mechanisms of GBM tumorigenesis are not clear. This leads to ineffective therapeutic intervention, and many patients relapse. However, with the current treatment options, i.e., surgery, radiotherapy, and chemotherapy, patient life expectancy can be increased, but these are not curative. To find the remedial solution, understanding the molecular features and identification of GBM subtypes is crucial. An earlier study shows that GBM can be classified into four subtypes based on transcriptional features, i.e., classical, neural, proneural, and mesenchymal. However, recent findings suggest that the neural subtype probably arises due to the contamination of normal neuronal tissue tumor margins (Wang et al., 2018). Therefore, GBM is currently classified into three subtypes. There are many other studies to find other subtypes using omics and clinical data (Park et al., 2019). Histopathological-based diagnosis is the most common method for subtype identification. However, it often leads to the inaccurate classification of subtypes due to interobserver variability (Van den Bent, 2010). Accurate pathological subtype diagnosis is pivotal for optimal patient management. Because GBM subtypes are histologically and genetically heterogeneous, they differ in gene expression, mutation, and epigenetic states, which lead to different therapeutic responses and clinical outcomes (Brennan et al., 2013; Zhang et al., 2020).

Recent advances of sequencing technologies have helped generate massive omics data in cancer, leading to a deep understanding of the molecular mechanisms in both common and rare cancers (Mardis and Wilson, 2009; Campbell et al., 2020). Data from sequencing experiments reveal that cancer initiation, progression, and maintenance are caused by the perturbations in multiple genomics and epigenomics factors. Additionally, genomics and epigenomics biomarkers have emerged as promising tools for developing the precision medicine and stratification of cancer subtypes and grades (Aran et al., 2013; Jurmeister et al., 2019; Jayanthi et al., 2020; Yoon et al., 2021). Alteration of gene expression and DNA methylation is the most prominent genomic and epigenomic event in cancer cells (Chakravarthi et al., 2016). The genome-wide analysis reveals that changes in gene expression and methylation patterns in several positions in the genome are strongly associated with GBM formation and progression (Bozdag et al., 2013; Dong and Cui, 2019; Vinel et al., 2021). Gene expression and methylation are both strongly interlinked processes; methylation levels in promoter regions influence the gene expression by regulating transcription factor binding (Mallik et al., 2020b). On many occasions, hypermethylation of CpG sites on promoter regions inhibits gene expression, whereas hypomethylation causes higher expression of genes (Moore et al., 2012). Therefore, classification using multiple “omics” data, i.e., transcriptome and methylome, can provide optimal features for the clinical diagnosis of cancer subtypes. However, enormous amounts of genetic and epigenetic alterations pose challenges to finding the unique molecular marker for diagnosing GBM subtypes. Benefitting from recent advances in computational methods, such as deep learning (DL) and traditional machine learning (ML), it is possible to scan the genome-wide transcriptome and methylome data to find the subtype-specific molecular feature for diagnosis (Qin et al., 2020).

We have implemented ML and DL algorithms for the precise and accurate classification of GBM subtypes in the present work. Each data type (i.e., transcriptome and methylome) and its integrated subsystem were separately used for classification. We found that the performance of the convolutional neural network (CNN) was superior (always >90%) compared with the other ML models. In addition, we examined the biological relevancy of features using weighted gene co-expression network analysis (WGCNA) and Gene Ontology (GO) analysis. Results show distinct co-expression modules are linked to each GBM subtype and are associated with subtype-specific biological functions. Moreover, several genes in the co-expression module are associated with patients’ survival. Overall, our findings suggest that a combination of LASSO feature selection and CNN can classify the subtype of GBM with higher accuracy and be used for clinical diagnosis.

## Materials and Methods

### Data Collection, Preprocessing, and Integration

In this study, we analyze TCGA GBM transcriptome (RNA-seq) and methylome (Illumina Infinium HumanMethylation450 platform) data. The data set was retrieved from UCSC Xena (https://xena.ucsc.edu/) (Goldman et al., 2020). Log2 (RSEM +1) (RSEM: RNA-Seq by Expectation Maximization) values for transcriptome, and β values for methylation were used for analysis. Next, the low-expression genes were removed from transcriptome data [log2 (RSEM +1) <0.1 in 90% sample], and data was scaled before analysis. Based on the clinical information, patients (*n* = 155) were divided into three categories based on cancer subtype, i.e., classical (*n* = 42), mesenchymal (*n* = 55), and proneural (*n* = 39) for transcriptome data. Similarly, based on the clinical information, we divided the methylome data (*n* = 84) into a particular subtype, i.e., classical (*n* = 29), mesenchymal (*n* = 32), and proneural (*n* = 23). Next, based on the clinical information, patients with both transcriptome and methylome profiles in TCGA were screened to integrate the transcriptome and methylome data. The total number of these patients with omics data was 52, including classical (*n* = 16), mesenchymal (*n* = 22), and proneural (*n* = 14). Due to the unavailability of healthy patient data for both transcriptome and methylome, we used the *Z*-score to classify higher and lower expression of genes and hyper- and hypo-methylated CpG sites. We calculated the Z-score for each gene or CpG site in a specific subtype using the following formula:

$$Z-score=\frac{\bar{x}-\mu }{\sigma }.$$

Here, $\bar{x}\;$ represents subtype-specific average expression or methylation level of a gene/CpG site, and µ and σ represent the population mean and population standard deviation, respectively (Bandyopadhyay et al., 2014). We applied *Z*-score>1 for higher expression and hypermethylation and *Z*-score < -1 for lower expression and hypomethylated on each subtype of GBM. Next, we screened the higher and lower expressed genes whose promoter regions were differentially methylated, considering that the differential methylation in the promoter regions may alter the corresponding gene’s expression. Finally, genes with both differential expression patterns and differential methylation promoter regions were used for further analysis (Maegawa et al., 2010; Sumithra et al., 2019). We collected the external data set from the Gene Expression Omnibus (GEO) repository for validation. GSE145645 was used to validate the model constructed using transcriptome and integrated data. GSE145645 contained all the subtypes of GBM, i.e., classical (*n* = 9), mesenchymal (*n* = 14) and proneural (*n* = 9). Models built on methylome data were further validated using GSE128654, which consisted of classical (*n* = 11), mesenchymal (*n* = 8), and proneural (*n* = 10) subtypes.

### Clustering Using t-SNE and Principal Component Analysis

The subtype-specific clustering of patients using transcriptome, methylome, and integrated data was visualized by t-distributed stochastic neighbor embedding (t-SNE) and principal component analysis (PCA) (Van der Maaten and Hinton, 2008). t-SNE was performed using the TSNE package in Python. For each t-SNE run, 1000 embeddings were created. Apart from that, we used PCA for better visualization of GBM subtypes; the ggfortify and cluster packages in R were used.

### Features Selection

We performed feature or variable selection to improve the performance of ML and DL algorithms. The least absolute shrinkage and selection operator (LASSO) was performed on all types of preprocessed data (Muthukrishnan and Rohini, 2016). We used default parameter values for lambda (the tuning factor that controls the strength of penalty) and dropped those genes having the coefficient value of zero. LASSO was implemented in the ScikitLearn (https://scikit-learn.org) package in Python.

### ML and DL Models for Classification of GBM Subtypes

We performed classification on the subtype of GBM as a multiclassification problem using gene expression levels as covariates. Several ML and DL algorithms were used for classification: support vector machine (SVM), random forest (RF), naïve Bayes (NB), logistic regression (LR), k-nearest neighbors (kNN), and CNN. SVM is used for the classification between the classes to find the optimal hyperplane (Afifi et al., 2017). The optimal hyperplane boundary not only separates the classes, but also maximizes the margin between the classes. The margin is the longest distance between the hyperplane and the nearest data (support vector) in each class. RF is a tree-based ensemble learning method that constructs several decision trees and gives the output for classification based on a majority vote between the estimators (trees). Gaussian NB classifier is an easy and simple Gaussian distribution that is dependent on the application of the Bayes theorem (Kaviarasi and Gandhi Raj, 2019). In Gaussian NB, each variable is considered as an independent variable and trained efficiently in supervised learning. It requires small measures of training data, which are essential for characterization and necessary for classification. A logistic regression classifier predicts the response based on one or more predictor variables. It measures the relationship between the categorical dependent variable and one or more independent variables by estimating probabilities using a logistic function. kNN (Liu et al., 2012) is a clustering algorithm that is widely used for pattern classification based on similarity measures. It utilizes standard Euclidean distance and evaluates the distinguishing features. kNN estimates the class attribute depending upon a neighborhood of close (or similar) patterns in the feature space. CNN is one of the deep feed-forward artificial neural network architectures that consist of the convolutional layer, activation function, and pooling layer. Convolution is one type of linear operation used instead of general matrix multiplication in convolution layers where filters are applied to original data or to feature maps in deep CNN. The convolution operation (denoted by an asterisk) is defined by

f(t)=(x∗K)(t),

where the function x (t)  is referred to as input, K(t) is referred to as kernel, and f(t) is referred to as output. In this paper, all ML classifiers on the Python platform use the sklearn library. The Keras library was used to construct the model architecture for CNN. Eight convolutional layers were used for obtaining the best result. All parameters for CNN are provided in Supplementary Table S1. Furthermore, parameters were optimized by the grid search method using the GridSearchCV package in Python. After obtaining optimal features, stratified k-fold was applied on the 70% training data set, and average performance measures were recorded. In stratified k-fold CV, the data set is divided into *k* independent folds, where *k*-1 folds were used to train the network, and the remaining one is reserved for test purposes. This procedure is then repeated until all folds are used once as a test set. The final output is then computed by averaging over the obtained performance parameters from each test set.

### Performance Evaluation

The performance of ML and deep learning models was evaluated using accuracy, recall, precision, F1-score, FPR, GM, and MCC. At first, we generated a confusion matrix to compute these performance scores. The confusion matrix is a table that categorizes the model’s prediction of whether it matches the actual value. We calculated true positive (TP), true negative (TN), false positive (FP), false negative (FN) from the confusion matrix (Mallik and Zhao, 2020). Then, we calculated the accuracy or success rate as

$$Accuracy=\frac{TP+TN}{TP+TN+FP+FN}.$$

The sensitivity or TP rate of an ML model was measured using the following equation:

$$Sensitivity=\frac{TP}{TP+FN}.$$

The specificity or TN rate of an ML model was measured using the following equation:

$$Specificity=\frac{TN}{TN+FP}.$$

The precision or positive predicted value was measured using the following equation:

$$Precision=\frac{TP}{TP+FP}.$$

A measure of model performance that combines precision and recall into a single number is known as the F measure or F1-score. The following equation was used to compute the F1-score:

$$F1-score=\frac{2\times TP}{2\times TP+FP+FN}.$$

Geometric mean (GM) is the average value or mean, which signifies the central tendency of the set of numbers by taking the *n*th root of the product of their values.

$$Geometric\;mean=\left(x_{1},x_{2}\ldots \ldots .x_{n}\right)\frac{1}{n}.$$

Mattews correlation coefficient (MCC) measures the correlation of the true classes with the predicted labels.

$$MCC=\frac{\left(TP*TN\right)-\left(FP*FN\right)}{\sqrt{\left(TP+FP\right)\left(TP+FN\right)\left(TN+FP\right)\left(TN+FN\right)}}.$$

We used the sklearn metrics library in Python to calculate the above score by importing functions such as confusion_matrix and classification_performance. Finally, we visualized the model performance across a wide range of conditions using receiver operating characteristic curve (ROC) plots using the roc_curve function.

### Ranking of the Model

Algorithm performance was compared using multi-criteria decision analysis (MCDA)/multi-criteria decision making (MCDM). The technique for order of preference by similarity to ideal solution (TOPSIS), an established MCDM method, was used to rank. Multiple criteria, such as accuracy, sensitivity, precision, G-mean, F-measure, FPR, and MCC, were used in TOPSIS (Triantaphyllou, 2000).

### Weighted Correlation Network Analysis

We identified co-expressed gene modules and analyzed the module-trait relationship using the WGCNA package in R (Langfelder and Horvath, 2008). First, the similarity matrix between each pair of feature genes in a specific subtype was measured based on Pearson’s correlation coefficient. Next, we transformed the similarity matrix into an adjacency matrix. The soft power β value was calculated for building the proximity matrix so that the co-expression network conformed to a scale-free network based on connectivity. Subsequently, we computed the topological overlap matrix (TOM) and the corresponding dissimilarity (1-TOM) value. Next, a dynamic tree cut algorithm was implemented to detect gene co-expression modules. The co-expression modules were constructed with a cut height of 0.6, and a minimum module size was set to 10 (transcriptome), 10 (methylome), and 5 (integrated) genes, respectively.

### Gene Set Enrichment and Survival Analysis

We performed the biological process and functional enrichment analysis using Enrichr (Kuleshov et al., 2016). Terms were considered statistically significantly enriched if the adjusted *p*-value was less than 0.05. The gene list from each positively correlated module was used to examine the enrichment of GO biological processes and molecular function terms. We performed overall survival and log-rank test of a co-expressed module using the survminer and survival package in R. We calculated the average expression of all genes in the module. Survival was compared between two groups: patients with higher (⋝75 percentile) and lower (⋜25 percentile) gene expression levels. Furthermore, we performed the overall survival analysis of specific genes using GEPIA (Tang et al., 2017). GEPIA performs survival analysis based on The Cancer Genome Atlas (TCGA) gene expression levels and patient clinical information. Here, the TCGA GBM data set was used for survival analysis. GEPIA generates Kaplan–Meier plots and performs the log-rank test to identify the genes associated with patient survival.

## Results

The etiology of GBM is associated with the alteration of transcriptome and methylome patterns. Therefore, the multi-omics approach that combines genome-wide methylation with transcriptome (RNA-seq) data can provide novel insights into biological function and disease mechanisms. In this work, we first separately analyzed the transcriptome and methylome, and then we integrated both data types to identify the molecular feature and classify the GBM subtypes.

### Classification of GBM Subtype Using Transcriptome

**FIGURE 1:**
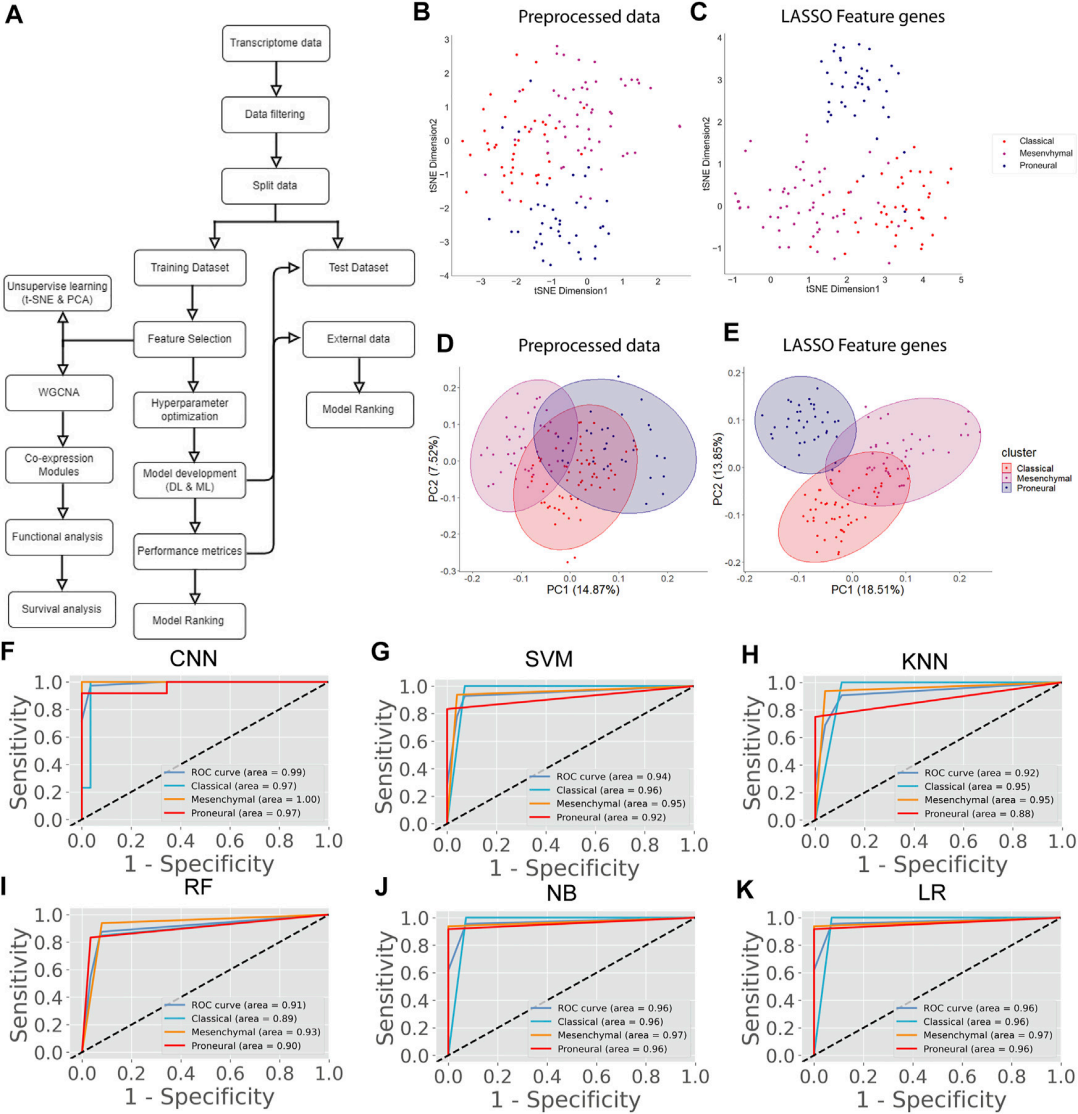
GBM subtype classification using transcriptome data. **(A)** The flow chart shows DL and ML pipelines using genome-wide transcriptome data to classify the subtypes. **(B, C)** t-SNE plots to visualize the subtype-specific clustering of the patients using preprocessed data and features genes. **(D, E)** PCA plots to visualize the subtype-specific clustering of patients using preprocessed data and feature genes. **(F–K)** ROC of various prediction models. ROC plots were generated using a test data set.

The transcriptome data of the GBM at TCGA contained 20,531 genes. After removing the low-expression genes, a total of 14,125 genes were found expressed in all GBM subtypes, including classical (*n* = 42), mesenchymal (*n* = 55), and proneural (*n* = 39). These genes were taken for further analysis. However, 14,125 genes could not be used as variables for prediction as the data is high-dimensional, leading to the inaccurate classification of subtypes. Therefore, we performed the LASSO to reduce the dimension of data and subsequently for selecting top key feature genes to enhance the prediction accuracy of the DL and ML model. LASSO performs *L*1 regularization and adds a penalty to the loss function. This penalty contains the absolute value of the regression coefficients. It attempts to minimize the cost function and automatically selects relevant features that are useful, and the remaining features are discarded with a coefficient equal to zero. The coefficients of the regression variables having nonzero values were selected as an optimal feature for further processing. A total of 201 feature genes were obtained after performing the LASSO analysis (Supplementary Table S2). Next, we performed t-SNE and PCA to examine the local structure of data, including 14,125 genes and 201 feature genes. We observed improved subtype-specific separation between patients using 201 feature genes compared to 14,125 genes, indicating that the LASSO feature selection method efficiently extracted most variable features from the transcriptome data (Figures 1B–E). Additionally, the percentage of variability in principal component 1 (PC1) was increased in the PCA of 201 feature genes compared with the preprocessed data (Figures 1D,E). These results indicate that information contained in 201 feature genes could separate the subtype with higher accuracy upon implementing DL and ML algorithms. However, distinct clusters of subtypes were not formed either in t-SNE or PCA.

Next, we proceeded to apply DL (CNN) and ML algorithms (i.e., SVM, KNN, RF, NB, LR) to classify subtypes of GBM using these feature genes as variables. We divided the data into training (70%) and test (30%) data sets. Seventy percent of the data was used for parameter optimization and to assess the performance of each model. The remaining 30% of data was used for independent predictors. Additionally, an external data set was also used for the final validation of models (Figure 1A). In the model training step, 70% of the data was used to obtain the best combination of hyperparameters using the grid search method for each DL and ML model. Next, we performed the stratified k-fold cross-validation (k = 10) on the training data using the optimal hyperparameters obtained from the grid search and recorded average performance measures of each model (Table 1). The performance of the models was evaluated using average accuracy, recall, precision, F1-score, FPR, GM, and MCC (see materials and methods). We observed that the prediction accuracy of CNN was superior (98.56%) compared with the other ML models. Even standard deviation (±0.03) and FPR (0.01) were minimum in the case of CNN. The MCC score is 0.97 for CNN, which represents the excellent correlation between the observed and predicted classifications. We observed that the performance of other ML classifiers was also good (accuracy >90%). Therefore, to compare the overall performance, we performed MCDM using TOPSIS (Si et al., 2021). All performance measures mentioned in Table 1 were considered for the ranking, and CNN topped the overall ranking. To validate this observation, we performed the classification using two data sets, i.e., 30% data as the test data (or independent data) and an external data set from GEO (GSE145645). In the test data, the prediction accuracy (98.56%) of CNN was superior to other ML models and the MCC score was 0.96 (Supplementary Table S3). It is always desirable to have a highly sensitive and highly specific model for diagnosis. Therefore, we visualized the relationship between sensitivity and specificity using the ROC curve (Figures 1F–K). The ROC curve represents the probability of a TP result or the test’s sensitivity against the probability of an FP result for a range of different cutoff points. Figure 1F shows the area under the ROC curve (AUC) is 0.99 for CNN, indicating that CNN can classify the GBM subtype with high specificity and sensitivity for clinical diagnosis. Additionally, classification with the external data set also represented a similar outcome; i.e., the performance of CNN was higher (Table 2). While validating with the external data set, we implemented tenfold cross-validation to calculate the average performance measure and compared the model performance by computing the rank. Furthermore, we compared the classification accuracy of the LASSO feature with the features selected using the variance. Gene with higher variance may contain more useful information. We selected the top 201 variable genes according to the degree of variance across all samples to compare the performance with LASSO. We performed the CNN using the same parameters and tenfold cross-validation. The average accuracy was 84.02% (±0.08). Therefore, the accuracy of prediction was less than LASSO features (98.56%). Hence, model building to validation, we observed that the feature genes from LASSO and CNN were the best for subtype classification for the transcriptome data. Therefore, we implemented this framework in subsequent analysis.

**TABLE 1:** Models performance and ranking for transcriptome data.

Method	Performance measures (Average of tenfold cross-validation)							MCDM Rank
	Accuracy	Recall	Precision	F1-score	FPR	GM	MCC	
SVM	91.42% (±0.08)	84.48	91.80	85.51	0.06	91.52	0.82	4
KNN	91.03% (±0.06)	85.78	90.59	86.06	0.07	91.44	0.82	5
RF	93.06% (±0.08)	88.52	93.04	89.15	0.05	93.02	0.85	3
NB	90.15% (±0.07)	86.08	87.16	85.38	0.08	90.52	0.80	6
LR	93.32% (±0.05)	89.47	92.12	89.97	0.05	93.61	0.86	2
CNN	98.56% (±0.03)	97.86	98.36	97.81	0.01	98.64	0.97	1

**TABLE 2:** Models performance and ranking for validation data (transcriptome).

Method	Performance measures (Average of tenfold cross-validation)							MCDM Rank
	Accuracy	Recall	Precision	F1-score	FPR	GM	MCC	
SVM	79.14% (±0.14)	71.33	63.57	65.68	0.11	84.07	0.71	4
KNN	79.15% (±0.14)	71.33	63.57	65.68	0.11	84.07	0.71	5
RF	80.57% (±0.22)	71.38	65.75	67.54	0.10	85.85	0.66	3
NB	77.59% (±0.17)	68.28	61.90	64.02	0.12	82.99	0.68	6
LR	81.20% (±0.15)	74.68	66.01	68.90	0.10	86.44	0.75	2
CNN	92.70% (±0.12)	90.20	88.77	89.24	0.01	98.25	0.96	1

### Classification of GBM Subtype Using Methylome

**FIGURE 2:**
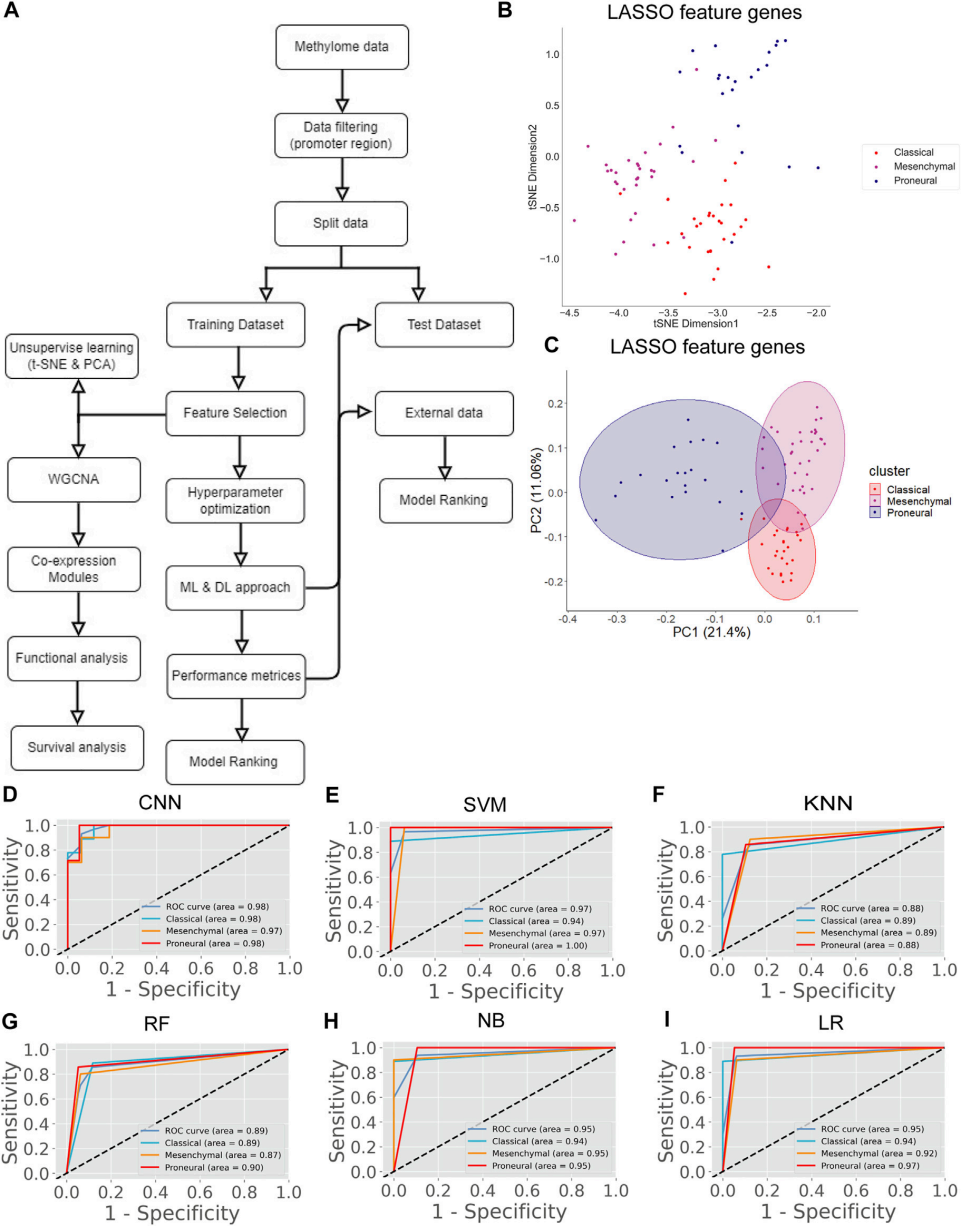
GBM subtype classification using methylome data. **(A)** The flow chart shows DL and ML pipelines using genome-wide DNA methylation data to classify the subtypes. **(B, C)** t-SNE and PCA plots to visualize the subtype-specific clustering of the patients from features gene. **(D–I)** ROC of various prediction models. ROC plots were generated using the test data set.

In the previous section, we classify the GBM subtype using the transcriptome data (or gene expression data) because the alteration of gene expression is a hallmark of oncogenesis. However, the level of gene expression is regulated by DNA methylation. Therefore, changes in DNA methylation patterns can play a crucial role in GBM development. Recent studies show that DNA methylation biomarkers are essential for improving and designing cancer therapy (Locke et al., 2019). Hence, the information contained in methylation data could possibly help to classify the GBM subtype. The genome-wide methylation or methylome data of 84 GBM patients were retrieved from the UCSC Xena database. We selected the data from the Illumina Infinium HumanMethylation450 platform (450K array) that has 4,85,577 probe sites. In this data set, the methylation level is estimated using the beta value. The beta value ranges from zero to one, representing the ratio of the intensity of the methylated bead type to the combined locus intensity. Thus, higher beta values represent a higher level of DNA methylation, i.e., hypermethylation and lower beta values represent a lower level of DNA methylation, i.e., hypomethylation. The recent reports show that the hypermethylation/hypomethylation level in the promoter region (e.g., defined as TSS1500 upstream to TSS200 downstream of TSS, 5′UTR, and first exon; TSS denotes transcription start site) and gene body determine the gene expression level (Sandoval et al., 2011; Yang et al., 2014). Therefore, we screened the promoter and gene body methylation data to perform classification because the alteration of methylation levels in these regions can influence the gene expression level and subsequently influence the biological processes (Dhar et al., 2021). The CpG sites, which include all promoter regions and the gene body, were screened for feature selection. By using LASSO, we obtained 498 features CpG sites (Supplementary Table S2). Next, we examined the subtype-specific clustering of patients with these 498 features CpG sites using t-SNE and PCA. Results show that there was slighter mixing among the different subtypes (Figure 2B,C). Next, we performed the DL and ML using these 498 CpG sites as variables. We repeated the same methodology as described in the previous section. First, the methylome data were divided into training (70%) and test (30%). The hyperparameters were optimized using the grid search method, and tenfold cross-validation was performed on the training data. The average performance measures were used to select the top-performing model using MCDM (Figure 2A). The overall performance of CNN was superior compared with other ML models using methylation data as well (Table 3). Next, we validated our observation with the 30% test data set (Supplementary Table S4) and an external data set (GSE128654) (Table 4). ROC plots (Figures 2D–I) show that the performance of the CNN (AUC = 0.98) was better compared with other ML models. However, the accuracy value is 89.0%, which is lower than the ML models. The overall performance of CNN on external data is superior (Rank = 1, see Table 4). These results indicate that CNN is the best classifier for predicting the GBM subtype using DNA methylation data.

**TABLE 3:** Models performance and ranking for methylation data.

Method	Performance measures (Average of tenfold cross-validation)							MCDM Rank
	Accuracy	Recall	Precision	F1-score	FPR	GM	MCC	
SVM	90.61% (±0.09)	86.40	87.67	84.49	0.07	90.55	0.81	4
KNN	90.72% (±0.12)	85.86	88.10	84.90	0.07	90.36	0.81	5
RF	91.03% (±0.10)	86.92	89.74	86.33	0.06	90.81	0.82	3
NB	92.34% (±0.08)	88.85	92.63	88.46	0.05	92.03	0.84	2
LR	89.84% (±0.11)	83.71	82.70	81.80	0.08	89.46	0.78	6
CNN	97.54% (±0.05)	96.77	97.71	96.47	0.01	97.47	0.95	1

**TABLE 4:** Models performance and ranking for external data (methylation).

Method	Performance measures (Average of tenfold cross-validation)							MCDM Rank
	Accuracy	Recall	Precision	F1-score	FPR	GM	MCC	
SVM	82.42% (±0.23)	76.65	73.58	74.60	0.09	85.26	0.76	4
KNN	79.09% (±0.20)	68.00	63.19	64.22	0.13	81.49	0.66	6
RF	82.81% (±0.16)	76.27	70.31	72.29	0.08	88.19	0.78	3
NB	81.52% (±0.15)	71.46	65.50	66.91	0.11	83.45	0.71	5
LR	87.42% (±0.17)	84.34	81.08	82.17	0.05	92.92	0.86	2
CNN	91.91% (±0.13)	90.50	89.15	89.60	0.01	97.63	0.96	1

### Classification of GBM Subtype by Integrating the Methylation and Transcriptome Data

**FIGURE 3:**
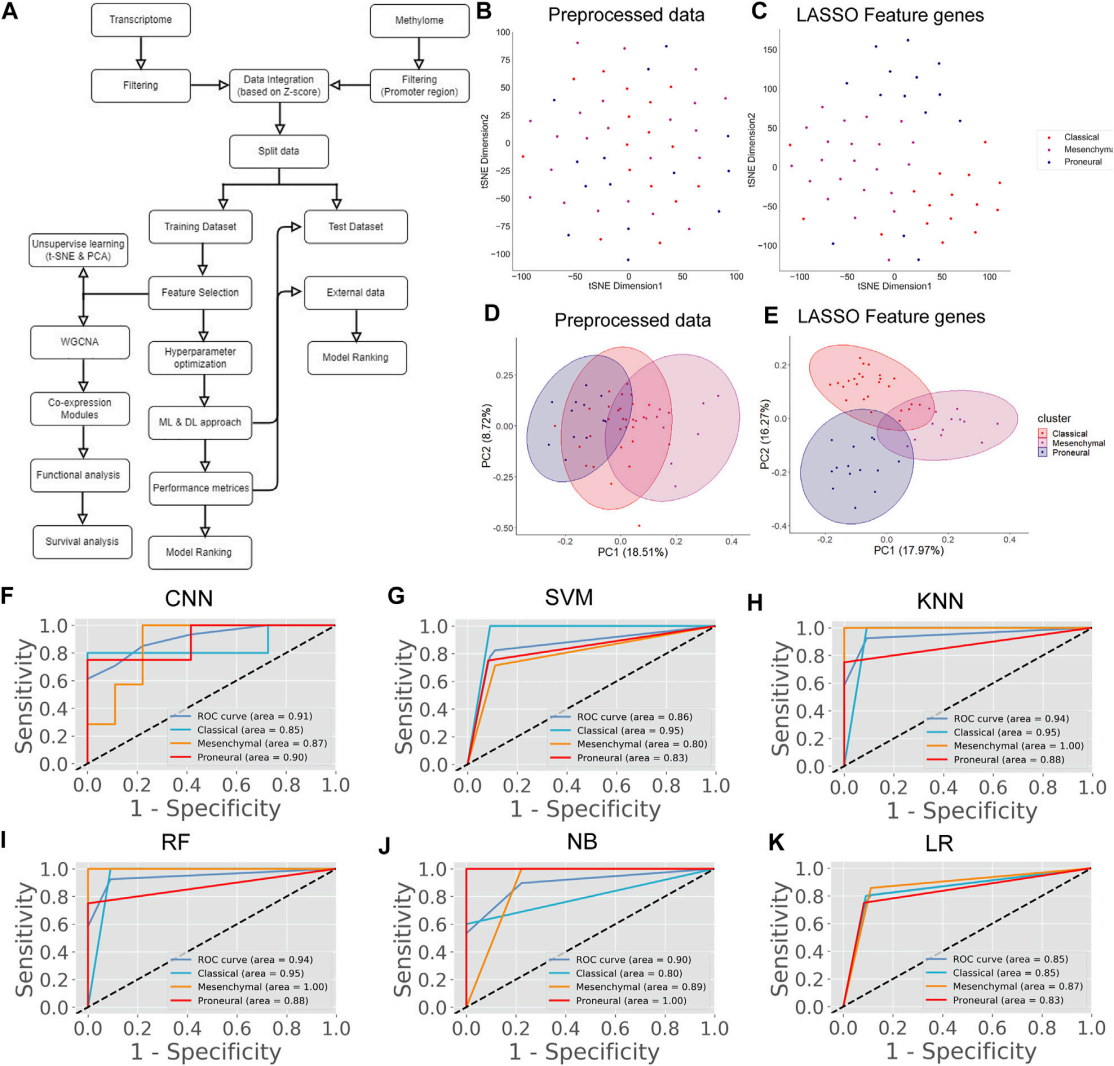
GBM subtype classification using integrated data. **(A)** The flow chart shows DL and ML pipelines using the integrated data of transcriptome and methylome to classify the subtypes. **(B,C)** t-SNE plots to visualize the subtype-specific clustering of patient from features gene. **(D,E)** PCA plots to visualize the subtype-specific clustering of patient from features gene. **(F–K)** ROC of various prediction models. ROC plots were generated using the test data set.

There are several studies where only one type of “omics” data is used, such as either gene expression or methylation data, to identify the biomarkers or classify the cancers (Díaz-Uriarte and Alvarez de Andrés, 2006; Wang et al., 2020). However, DNA methylation and gene expression are integrated processes that determine cellular fate (Basu and Tiwari, 2021). The perturbation of gene expression in many human cancers is due to the change of methylation pattern (Langfelder and Horvath, 2008). Hence, integrating these strongly interlinked cellular processes and subsequent analysis could facilitate finding a more effective diagnostic option (Mallik et al., 2020a). The patients having both transcriptome and methylome data were selected for data integration. Next, we screened the gene and methylation sites based on *z*-score, i.e., *z* > 1 and *z* < -1 (see materials and methods). A *z*-score greater than 1 or less than -1 indicates the expression and methylation is greater or less than the population mean. We identified common genes whose expression and methylation both are *z* > 1 or *z* < -1 in each subtype. Next, we combined all these genes (*n* = 4,231) and used their gene expression level to find the most variable features (*n* = 75) using LASSO (Supplementary Table S2). We observed that 75 feature genes form the distinct subtype-specific clusters with PCA and t-SNE (Figure 3B–E). Compared with previous features from transcriptome and methylome data, the feature genes of the integrated data significantly improve the clustering of the GBM subtype. Next, we implemented CNN using these feature genes and compared CNN performance with the other five ML algorithms (Figure 3A). In this case, the CNN performance was also ranked on top (Table 5). Furthermore, we validated the model with 30% test data (Supplementary Table S5) and external data (Table 6). ROC plots generated using test data explain the decent performance of CNN (AUC = 0.91 and accuracy = 87.50%) (Figures 3F–K). The validation with external data showed that CNN was the top performer (accuracy = 94.48%) for classification (Table 6). It can be concluded that in all three types of analysis, CNN efficiently classified the GBM subtypes. However, the features from integrated data specifically cluster the subtype of GBM with PCA and t-SNE. Moreover, the consistent all-around performance of CNN proves that CNN can be used as a computational tool for the clinical diagnosis GBM subtype.

**TABLE 5:** Models performance and ranking for integrated data.

Method	Performance measures (Average of tenfold cross-validation)							MCDM Rank
	Accuracy	Recall	Precision	F1-score	FPR	GM	MCC	
SVM	89.94% (±0.10)	86.47	81.11	81.65	0.07	90.02	0.82	5
KNN	91.87% (±0.13)	88.35	82.68	84.57	0.06	91.81	0.84	3
RF	93.67% (±0.10)	88.70	84.63	86.06	0.04	93.52	0.89	2
NB	89.95% (±0.14)	83.16	77.12	79.14	0.08	89.43	0.79	6
LR	92.18% (±0.10)	87.10	81.38	83.43	0.06	91.77	0.85	4
CNN	98.20% (±0.05)	98.44	97.97	97.60	0.01	98.25	0.97	1

**TABLE 6:** Models performance and ranking for external data (transcriptome).

Method	Performance measures (Average of tenfold cross-validation)							MCDM Rank
	Accuracy	Recall	Precision	F1-score	FPR	GM	MCC	
SVM	63.15% (±0.12)	46.43	35.70	37.89	0.22	68.38	0.38	6
KNN	67.08% (±0.17)	49.56	38.83	42.39	0.20	72.31	0.39	5
RF	80.00% (±0.19)	72.24	66.21	67.70	0.09	85.81	0.73	2
NB	66.14% (±0.17)	55.59	45.69	48.47	0.22	71.35	0.43	4
LR	70.74% (±0.10)	49.26	37.11	41.14	0.16	75.89	0.48	3
CNN	94.48% (±0.11)	94.48	94.48	94.48	0	1	1	1

### The Biological Relevance of Features and Identification of Biomarkers

**FIGURE 4:**
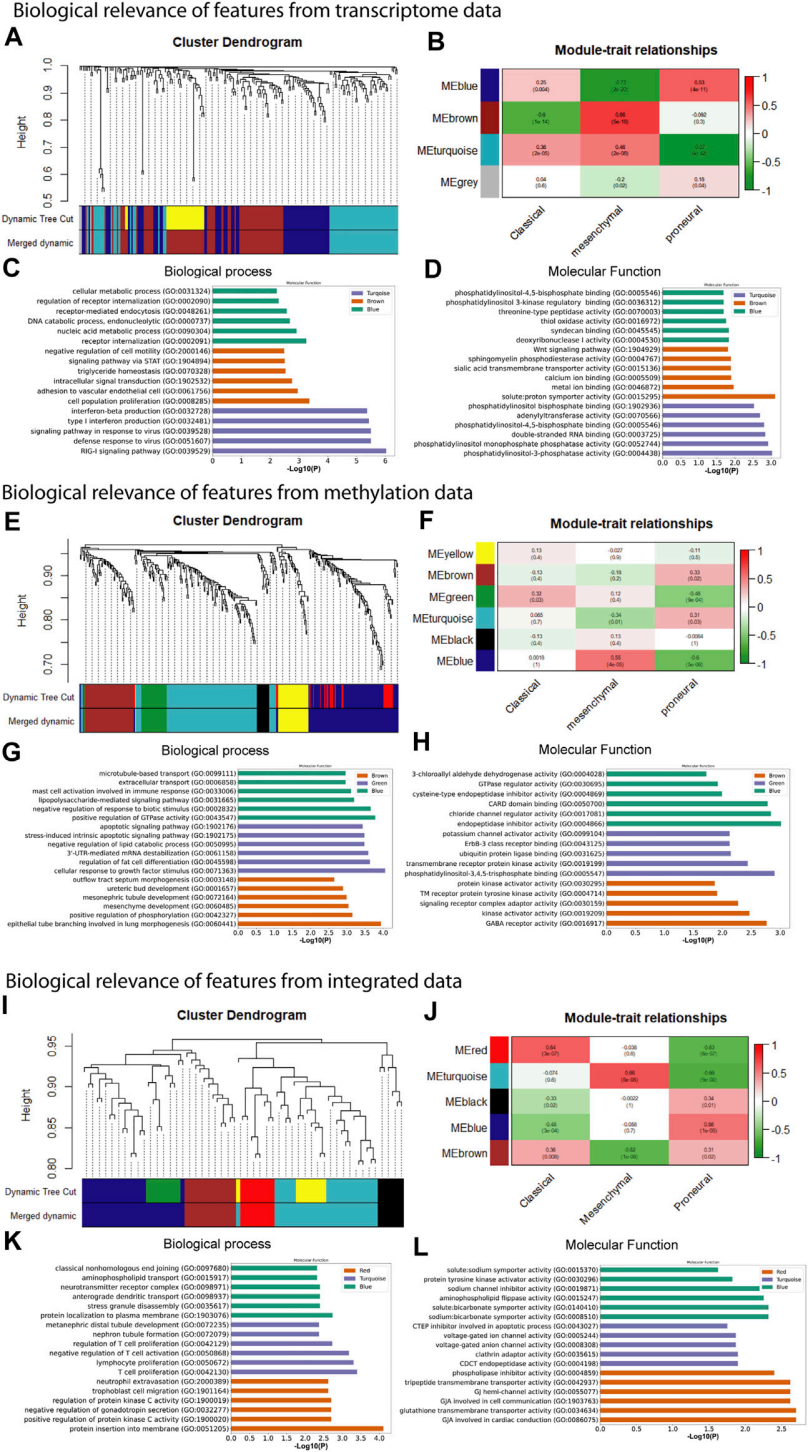
Weighted gene co-expression network analysis and gene set enrichment of feature used for model building. **(A)** co-expression gene module, **(B)** module-trait relationship, **(C)** biological process, and **(D)** molecular function of feature from transcriptome data. **(E)** co-expression gene module, **(F)** module-trait relationship, **(G)** biological process, and **(H)** molecular function of feature from methylome data. **(I)** co-expression gene module, **(J)** module-trait relationship, **(K)** GO biological process term analysis, and **(L)** GO molecular function of feature from integrated data.

In the preceding steps, we extracted features from large-scale transcriptome and methylome data sets to develop the predictive tool for subtype identification. We observed that selected features from each type of data have excellent separability power, and therefore, we achieved classification accuracy >90% in every case. This indicates that any subset of these features is probably associated with a particular subtype (or phenotype). Therefore, further analysis of these features genes can link the genotype to phenotype. We performed WGCNA to understand genotype-to-phenotype relationships. WGCNA can find the module of highly correlated genes and their association with a specific subtype of GBM (Langfelder and Horvath, 2008). We constructed the co-expression module using the feature genes expression from transcriptome, methylome, and integrated data and examined their association with specific subtypes. To find the co-expression module of feature methylation sites, we mapped the methylation site to gene name and extracted the gene expression data to construct co-expression modules. To construct the co-expression modules, we determined the soft threshold, β (*β* = 4, 6, and 5 for transcriptome, methylome, and integrated data, respectively) based on scale independence and mean connectivity (Supplementary Figure S1). We then merged modules with similarities above 0.6 for all three types of data. Finally, the dynamic tree cut showed a gene cluster dendrogram containing 3, 6, 5 co-expression models in the features of transcriptome, methylome, and integrated data, respectively (Figures 4A,E,I). To understand the genotype–phenotype relationship, we generated the module–trait relationship plot. We found distinct patterns of association between modules and subtypes (Figures 4B,F,J). Results show that the blue module (Figure 4B) was significantly and positively associated with the proneural subtype (*r* = 0.53, *p* = 4E-11). In contrast, it was negatively associated with the mesenchymal (*r* = -0.73, *p* = 2E-23), and weakly correlated with the classical subtype (*r* = 0.25, *p* = 0.004). Similarly, we found a distinct pattern of association between other modules (i.e., brown and turquoise) and subtypes (Figure 4B). We observed the same in the features from the methylome and integrated data. In methylome (Figure 4F), the brown module significantly and positively associated with only the proneural subtype (*r* = 0.33, *p* = 0.02). The green module is positively associated with the classical (*r* = 0.32, *p* = 0.03) and negatively associated with the proneural (*r* = -0.46, *p* = 9E-04). The blue module is strongly and positively correlated with the mesenchymal subtype (*r* = 0.55, *p* = 4E-05), whereas it was negatively associated with proneural (*r* = -0.6, *p* = 5E-06). However, the feature from the integrated data showed a more specific module–subtype association. At least one module was strongly and positively correlated with a specific subtype. The red (*r* = 0.64, *p* = 3E-07), turquoise (*r* = 0.66, *p* = 8E-08), and blue (*r* = 0.56, *p* = 1E-05) were explicitly and positively associated with classical, mesenchymal, and proneural, respectively (Figure 4J). The module–trait relationship analysis indicates that integration of transcriptome and methylome results in subsets of features strongly correlated with a particular subtype of GBM. Probably, the integrated data sets are mechanistically more relevant as the methylation and gene expression are integrated cellular processes. Next, we performed the gene set enrichment analysis (GSEA), i.e., GO biological process (BP) and molecular function (MF), using Enrichr to understand the biological relevance of each data type’s top three positively correlated modules (Mallick et al., 2020). We observed that modules were significantly (adjusted *p* < .05) associated with several BP and MF that are linked to oncogenesis. For example, the turquoise module from the transcriptome data in the classical subtype is involved in the RIG-I signaling pathway that elicits RIG-I-like receptors’ expression and activity (RLRs) (Figure 4C). These receptors stimulate both innate and adaptive immune responses against tumor antigens and promote the apoptosis of cancer cells (Bufalieri et al., 2021). In contrast, the brown module associated with the mesenchymal subtype (leukocyte adhesion to vascular endothelial cell) may be linked to the GBM-associated with the endothelial cell, that is, resistant to cytotoxic drugs, and also less apoptotic than healthy cells (Charalambous et al., 2006) (Figure 4C). Phosphatidylinositol 3 phosphate activity enriched in the turquoise module, solute proton symporter activity in the brown module, and syndecan binding in the blue module are associated with higher tumor grades and poor prognosis in GBM (Shi et al., 2017) (Figure 4D). Similarly, we observed that the blue module in the mesenchymal and the brown module in the proneural are linked to positive regulation of GTPase activity and positive regulation of phosphorylation in methylome data (Figure 4G). These processes are signatures of GBM formation and progression (He et al., 2021). Even molecular functions of several co-expression modules are involved in tumorigenesis, such as phosphatidylinositol 3, 4, 5 triphosphate binding enriched in the green module deregulates many key signaling pathways involving growth, proliferation, survival, and apoptosis in GBM (Mao et al., 2012) (Figure 4H). Furthermore, endopeptidase inhibitor activity, GABA receptor activity enriched in blue and brown modules, respectively, are predominant events in GBM (Labrakakis et al., 1998; Lin et al., 2020) (Figure 4H). The gene co-expressed modules in the integrated data, i.e., and the turquoise module (mesenchymal) involved with negative regulation of T cell activation and proliferation is one of the signatures of GBM (Woroniecka et al., 2018). The MF of the same module shows it is associated with gap junction channel activity involved in cell communication, which is also linked to GBM (Aasen et al., 2016) (Figures 4K,L).

**FIGURE 5:**
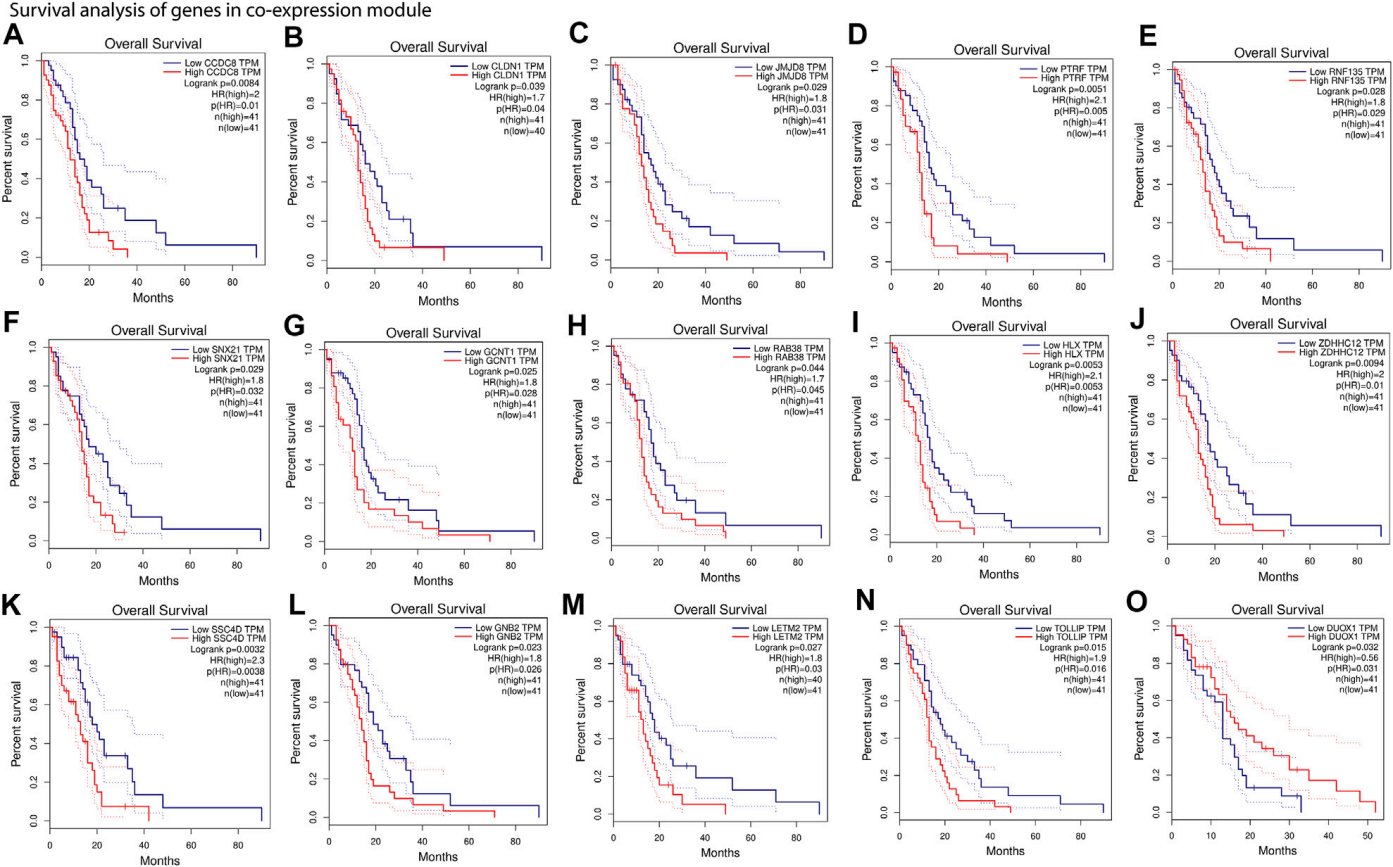
Survival analysis of gene present in co-expression module. **(A–F)**, Kaplan–Meier plots of genes from positively associated modules with the classical subtype. **(G–M)**, Kaplan–Meier plots of genes from positively associated modules with the mesenchymal subtype. **(N,O)**, Kaplan–Meier plots of genes from positively associated modules with the proneural subtype. Overall survival was analyzed based on the clinical information of the patients from TCGA and quartile method of 75% cutoff of higher and 25% cutoff of lower limit (An extended version of this figure is provided in Supplementary Figure S3).

Our results show that most of the positively correlated modules in GBM subtypes were involved in several BP and MF. Besides this, many of these BP and MF are involved in oncogenic processes. This shows a possibility of identifying these modules’ genes as cancer biomarkers for therapy or diagnosis. We performed survival analysis of positively correlated modules (Supplementary Figure S2). The turquoise module in the integrated feature is significantly (log-rank test, *p* = .029) associated with the patient survival. Hence, we performed survival analysis of all genes separately present in these modules using GEPIA web tools (Figure 5 and Supplementary Figure S3). We found several genes that were present in the co-expression module and also associated with the patient’s survival (log-rank test, *p* < .05). The higher expression of most of the genes was associated with worse survival of the patients, except DUOX1 (FIGURE 5O) and FOXN2 (Supplementary Figure S3). However, higher or lower expression of genes associated with worse survival can be considered biomarkers (Sun et al., 2019; Liu et al., 2021). Furthermore, several experimental articles confirm the involvement of these genes in GBM formation and progression. For example, CCDC8, CLDN1, JMJD8, PTRF, RNF135, and SNX21 in classical (Berezovsky et al., 2014; Karnati et al., 2014; Pangeni et al., 2015; Yeo et al., 2016; Huang et al., 2018; Zhang et al., 2019) (Figure 5A–F); GCNT1, RAB38, HLX, ZDHHC12, SRCRB4D (SSC4D), GNB2, and LETM2 in mesenchymal (Thaker et al., 2009; Chen et al., 2014; Toton et al., 2018; Chen et al., 2020; Bianchetti et al., 2021; Giambra et al., 2021; Katsushima et al., 2021) (Figure 5G–M); and TOLLIP and DUOX1 (Humbert-Claude et al., 2016; Little et al., 2016) in proneural (Figure 5N–O) are linked to GBM patient survival. The association of genes from the modules with patient survival shows the possibility to identify them as subtype-specific prognostic biomarkers. We also observed that the expression pattern of survival-associated genes varied across the subtype (Supplementary Figure S4). Furthermore, we illustrated with gene enrichment analysis that their biological process and molecular functions are also linked to oncogenic events. Therefore, these findings confirm the clinical validity of our models and can provide insight into the complex regulatory processes in different subtypes of GBM.

## Discussion

The present study indicates that DL and ML can be powerful tools for finding patterns in large-scale genetic and epigenetic data sets related to human cancer. In general, efficient DL and ML tools work like a black box; researchers or clinicians may not be confident in diagnosing or classifying cancer patients using these approaches. However, if the basis of classification is biologically relevant and has higher accuracy, the diagnosis and patient management are more assured and systematic. Here, we present a biologically relevant DL- and ML-based framework to classify the subtype of GBM to increase accuracy in diagnosis; in turn, it can lead to better patient management. Previous studies try to develop the cancer classification model using a single type of omics data. Models are mainly developed for binary classification to identify healthy and cancer patients. However, we use two types of high-throughput data, i.e., transcriptome and methylome; integrated forms of these data were explored to develop the classification framework. Most importantly, we successfully separate three subtypes, classical, mesenchymal, and proneural, of GBM. Although we dealt with multiclass classification problems, we still achieved classification accuracy >90%. We also compared DL and ML techniques to identify the most suitable method for interpreting the transcriptome, methylome, and integrated data. The DL method, i.e., CNN, outperforms other ML models. Using CNN, we were able to classify the tumor into the correct subtype from the test and external cohort. We observed that overall classification performance was higher using the transcriptome and integrated data than the methylome data.

Another significant aspect of our findings is the biological relevance of features and the identification of subtype-specific prognostic biomarkers. To find the association of feature genes with specific subtypes, we performed WGCNA. The gene co-expression module-subtype relation analysis revealed how a subset of features is strongly and positively correlated with a particular subtype of GBM. In addition to that, the gene set enrichment analysis revealed that all positively correlated modules are biologically relevant even those that are linked to oncogenic processes. Among all data types, a strong module–trait relationship was observed in feature genes from integrated data. Furthermore, we identified several genes present in these co-expressed modules, which were linked to patient survival. Our study explained how the feature genes from the DL/ML framework could be used to find the subtype-specific biomarkers. Good agreement was found when comparing prognostic markers from this work against published experimental data. The feature genes of this study and CNN can provide assured and clinically relevant deep learning-based diagnostic tools for the proper treatment of GBM patients. Furthermore, the results of this work unravel and shed light on the understanding of genotype-phenotype relationships of the GBM subtype. Last, much of the research presented in this work can be applied to other human cancers to design DL-based diagnostic tools using high-throughput experimental data.

## Data Availability

Publicly available data sets were analyzed in this study. This data can be found here: Cancer patient transcriptome data is available at https://xenabrowser.net/datapages/?dataset=TCGA.GBM.sampleMap%2FHiSeqV2&amp;host=https%3A%2F%2Ftcga.xenahubs.net&amp;removeHub=https%3A%2F%2Fxena.treehouse.gi.ucsc.edu%3A443, and methylome data is available at https://xenabrowser.net/datapages/?dataset=TCGA.GBM.sampleMap%2FHumanMethylation450&amp;host=https%3A%2F%2Ftcga.xenahubs.net&amp;removeHub=https%3A%2F%2Fxena.treehouse.gi.ucsc.edu%3A443. The validation data GSE145645 is available at https://www.ncbi.nlm.nih.gov/geo/query/acc.cgi?acc=GSE145645, and GSE128654 is available at https://www.ncbi.nlm.nih.gov/geo/query/acc.cgi?acc=GSE128654.
