# Supplementary material for: A Deep Learning–Based Framework for Supporting Clinical Diagnosis of Glioblastoma Subtypes
Source: Front Genet. 2022 Mar 28;13:855420. doi: 10.3389/fgene.2022.855420 (PMC9000988; doi:10.3389/fgene.2022.855420)
Supplement: Supplementary file 1 [file Table5.PDF]

Supplementary Table 5. Models performance and AUC from test data (integrated)

Method	Performance measures (on test dataset)							
	**Accuracy**	**Recall**	**Precision**	**F1-score**	**FPR**	**GM**	**MCC**	**AUC**
SVM	90.63	82.50	93.06	85.16	0.06	91.48	0.82	0.9
KNN	88.28	84.82	81.25	82.56	0.09	87.35	0.71	0.86
RF	87.50	80.54	80.54	80.54	0.09	87.50	0.71	0.85
NB	95.70	93.75	92.71	92.45	0.04	95.79	0.90	0.94
LR	95.70	93.75	92.71	92.45	0.04	95.79	0.90	0.94
CNN	87.50	78.75	84.31	79.01	0.10	87.50	0.72	0.91
