# Supplementary material for: A Deep Learning–Based Framework for Supporting Clinical Diagnosis of Glioblastoma Subtypes
Source: Front Genet. 2022 Mar 28;13:855420. doi: 10.3389/fgene.2022.855420 (PMC9000988; doi:10.3389/fgene.2022.855420)
Supplement: Supplementary file 3 [file Table4.PDF]

Supplementary Table 4. Models performance and AUC from test data (methylation)

Method	Performance measures (on test dataset)							
	**Accuracy**	**Recall**	**Precision**	**F1-score**	**FPR**	**GM**	**MCC**	**AUC**
SVM	97.19	95.73	96.85	96.09	0.02	97.42	0.94	0.97
KNN	89.94	84.15	86.98	84.86	0.07	89.73	0.77	0.88
RF	89.50	84.96	84.62	84.62	0.08	89.73	0.76	0.89
NB	95.12	92.26	94.02	92.55	0.02	94.85	0.89	0.95
LR	94.82	92.26	93.17	92.48	0.03	94.85	0.88	0.95
CNN	89.50	85.38	86.54	84.55	0.08	89.73	0.78	0.98
