# Supplementary material for: A Deep Learning–Based Framework for Supporting Clinical Diagnosis of Glioblastoma Subtypes
Source: Front Genet. 2022 Mar 28;13:855420. doi: 10.3389/fgene.2022.855420 (PMC9000988; doi:10.3389/fgene.2022.855420)
Supplement: Supplementary file 4 [file Table1.PDF]

Supplementary Table 1. Parameters of CNN

**Parameters**	**Datasets**		
	**Transcriptome**	**Methylome**	**Integrated**
Activation	relu	relu	relu
Batch_size	50	50	50
Dropout_rate	0.1	0.1	0.1
epochs	100	100	100
filters	32	1	3
Init_mode	uniform	uniform	uniform
Kernel_size	5	3	3
optimizer	RMSprop	RMSprop	Adam
