# Supplementary material for: A Deep Learning–Based Framework for Supporting Clinical Diagnosis of Glioblastoma Subtypes
Source: Front Genet. 2022 Mar 28;13:855420. doi: 10.3389/fgene.2022.855420 (PMC9000988; doi:10.3389/fgene.2022.855420)
Supplement: Supplementary file 5 [file Image4.PDF]

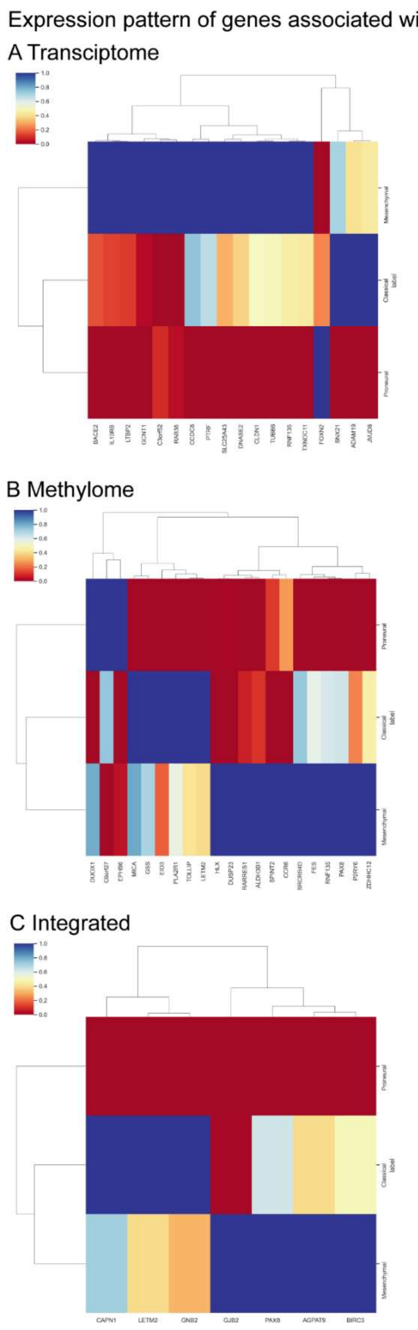


**Supplementary Figure 4.** Expression pattern of genes associated with patient survival. Heatmaps show the genes present in coexpressed modules of (A) transcriptome (B) methylome (C) integrated data
