# Supplementary material for: A Deep Learning–Based Framework for Supporting Clinical Diagnosis of Glioblastoma Subtypes
Source: Front Genet. 2022 Mar 28;13:855420. doi: 10.3389/fgene.2022.855420 (PMC9000988; doi:10.3389/fgene.2022.855420)
Supplement: Supplementary file 6 [file Image2.PDF]

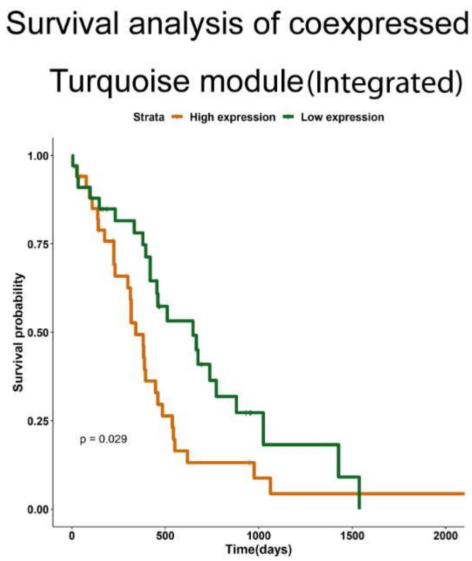

Supplementary Figure2 Survival analysis of positively associated module. Overall survival was analyzed based on quartile method of 75 % cut-off of higher and 25% cut-off of lower limit.
