# Supplementary material for: A Deep Learning–Based Framework for Supporting Clinical Diagnosis of Glioblastoma Subtypes
Source: Front Genet. 2022 Mar 28;13:855420. doi: 10.3389/fgene.2022.855420 (PMC9000988; doi:10.3389/fgene.2022.855420)
Supplement: Supplementary file 7 [file Image3.PDF]

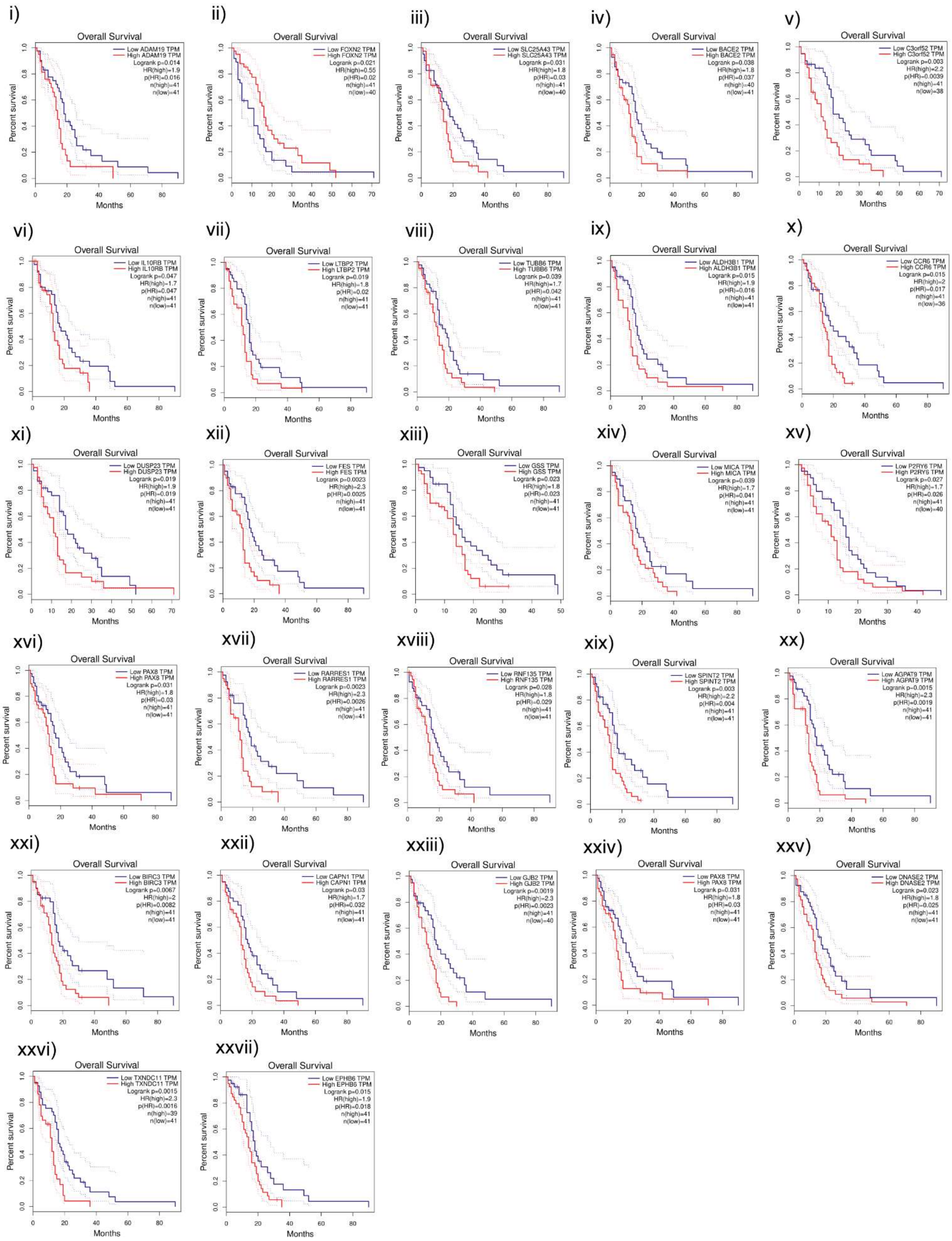

Supplementary Figure3 (Extended figure of Figure 5) Survival analysis using GEPIA of gene present in co-expression module. i to iii, Kaplan-Meier plots of genes from positively associated modules with classical subtype. iv to xxiv, Kaplan-Meier plots of gene from positively associated modules with mesenchymal subtype. xxv to xxvii, Kaplan-Meier plots of gene from positively associated modules with proneural subtype. Overall survival was analyzed based on quartile method of 75 % cut-off of higher and 25% cut-off of lower limit.
