# Supplementary material for: A Deep Learning–Based Framework for Supporting Clinical Diagnosis of Glioblastoma Subtypes
Source: Front Genet. 2022 Mar 28;13:855420. doi: 10.3389/fgene.2022.855420 (PMC9000988; doi:10.3389/fgene.2022.855420)
Supplement: Supplementary file 8 [file Table3.PDF]

Supplementary Table 3. Models performance and AUC from test data (transcriptome)

Method	Performance measures (on test dataset)							
	**Accuracy**	**Recall**	**Precision**	**F1-score**	**FPR**	**GM**	**MCC**	**AUC**
SVM	95.12	93.14	92.82	92.57	0.04	95.12	0.89	0.94
KNN	93.46	90.70	90.70	89.80	0.05	93.49	0.85	0.92
RF	91.73	87.14	87.61	87.30	0.06	91.86	0.81	0.91
NB	96.61	95.58	94.80	94.92	0.02	96.74	0.92	0.96
LR	96.61	95.58	94.80	94.92	0.02	96.74	0.92	0.96
CNN	98.33	97.56	97.21	97.28	0.01	98.37	0.96	0.99
