# Supplementary material for: A Deep Learning–Based Framework for Supporting Clinical Diagnosis of Glioblastoma Subtypes
Source: Front Genet. 2022 Mar 28;13:855420. doi: 10.3389/fgene.2022.855420 (PMC9000988; doi:10.3389/fgene.2022.855420)
Supplement: Supplementary file 9 [file Image1.PDF]

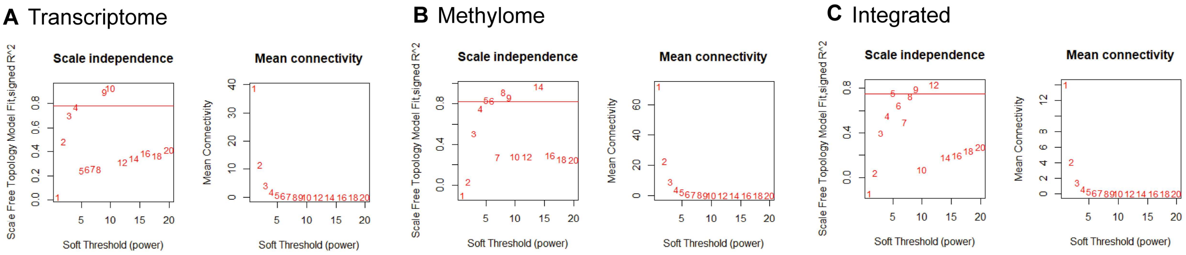

Supplementary Figure1. Analysis of network topology for several soft thresholding power (β) in WGCNA
